# Systematics of the genus *Zinaida* Evans, 1937 (Hesperiidae: Hesperiinae: Baorini)

**DOI:** 10.1371/journal.pone.0188883

**Published:** 2017-11-30

**Authors:** Jing Tang, Zhenfu Huang, Hideyuki Chiba, Yuke Han, Min Wang, Xiaoling Fan

**Affiliations:** 1 Department of Entomology, College of Agriculture, South China Agricultural University, Guangzhou, China; 2 B.P. Bishop Museum, Honolulu, Hawaii, United States of America; National Cheng Kung University, TAIWAN

## Abstract

Traditionally, species of the genus *Zinaida* were assigned to the genus *Polytremis*, until molecular evidence revealed that the former is a distinct genus. Nine species in *Polytremis* sensu Evans have since been removed and assigned to *Zinaida*; however, there is still uncertainty as to the taxonomic status of an additional seven *Polytremis* species. Moreover, the interspecific relationships within *Zinaida* have remained unresolved. To further investigate the taxonomic statuses and interspecific relationships within *Zinaida*, a molecular phylogeny of most species of *Zinaida* and its allies was inferred based on regions of the mitochondrial *COI-COII* and *16S* and nuclear *EF-1α* genes (3006 bp). The results revealed that *Zinaida* is monophyletic and consists of four intra-generic clades that correspond to morphological characteristics. Clade A (*Z*. *suprema* group) consists of *P*. *kiraizana*, *Z*. *suprema*, and *P*. *gigantea*, with the latter two as sister species. Clade B (*Z*. *nascens* group) consists of seven species, and is the sister group of Clade C (*Z*. *pellucida* group), which comprises sister species *Z*. *pellucida* and *Z*. *zina*. In Clade B, *Z*. *caerulescens* and *Z*. *gotama*, and *Z*. *theca* and *Z*. *fukia* are sister species, respectively. On the basis of our molecular evidence and morphological features, we have moved *P*. *gigantea*, *P*. *kiraizana*, *P*. *jigongi*, and *P*. *micropunctata* to the genus *Zinaida* as new combinations. We review morphological characteristics and discuss the distribution of each of these groups in the light of our phylogenetic hypothesis, and provide a comprehensive taxonomic checklist.

## Introduction

Recently, the molecular phylogeny of the tribe Baorini, particularly that of the genus *Polytremis* sensu Evans (1949), has attracted researchers’ attention [1–3]. Jiang et al. [2] treated *Polytremis* as a monophyletic genus. In their tree, all the “*Polytremis*” taxa were nested within a single clade, and *P*. *lubricans*, the type of *Polytremis*, was placed at the distal part of the tree with *eltola* and, then, those two taxa with *discreta*. Zhu *et al*. [3] indicated polyphyly of *Polytremis*, where *lubricans* was far apart from the rest of the taxa, but no taxonomic change was made. Fan *et al*. [1] insisted that the group should be polyphyletic, and divided it into three genera, *Polytremis*, *Zinaida*, and *Zenonoida*. Accordingly, *Polytremis* was located separately from *Zinaida* and *Zenonoida*. Despite the weak support, the relationship of *Polytremis* to the clade *Gegenes* and *Borbo* is closer than their relationship to *Zinaida* and *Zenonoida*. On the other hand, *Zenonoida* nested with *Zenonia* and *Zinaida*, and *Zenonia* + *Zenonoida* as sister to *Zinaida*, whereas Jiang *et al*. [4] preserved the monophyly of *Polytremis*. Of the 12 species Fan *et al*. analyzed, two (*P*. *discreta* and *P*. *eltola*) were assigned to the newly described *Zenonoida*, whereas nine were moved to the reinstated genus *Zinaida*. The only species remaining in *Polytremis* was its type, *P*. *lubricans*. As a consequence, the taxonomic status of seven species of *Polytremis sensu* Evans (1949) was left uncertain.

On the basis of the results of a preliminary morphological analysis conducted as part of the present study, we suggest that *P*. *gigantea* Tsukiyama, Chiba & Fujioka, 1997; *P*. *micropunctata* Huang, 2003; *P*. *jigongi* Zhu, 2012; and *P*. *kiraizana* Sonan, 1938 should be included in the genus *Zinaida*. Our first aim in the present study was to clarify the taxonomic status of these species based on molecular data.

In addition to the aforementioned uncertainty at the generic level, the interspecific relationships within the genus *Zinaida* also remain unresolved. In previous studies [1–3], the sister relationship between *Z*. *zina* and *Z*. *pellucida* has been the only matter of consensus, whereas the other interspecific relationships have remained uncertain. Accordingly, our second aim in the present study was to investigate the interspecific relationships within *Zinaida*, and then review the morphological characteristics of these species in light of our phylogenetic hypothesis and provide a comprehensive taxonomic checklist.

## Materials and methods

### Taxon sampling

A total of 17 specimens were examined in this study, seven of which (two *Zinaida* and five *Polytremis*, sensu Evans) were newly sampled as ingroup taxa (S1 Table). One of these specimens (He324) appeared to have the male stigma of *P*. *micropunctata* and male genitalia of *Z*. *nascens*. The specimen was identified as *Z*. *nascens* (S1 Fig), which indicates that a difference in the male stigma should not be considered a definitive taxonomic character. Samples of *P*. *micropunctata*, *P*. *minuta*, *P*. *annama*, and *P*. *kittii* could not be obtained, and thus their taxonomic status remains unresolved.

### DNA extraction, amplification, and sequencing

Samples of *Z*. *matsuii* (He1050), *P*. *gigantea*, and Z. *nascens* (He324) were analyzed afresh. The specimens used for DNA analysis were collected as adults and either preserved in 95% ethanol or dried. Total genomic DNA was extracted from dried legs or thoracic tissue using a Hipure Insect DNA Kit (Magen Inc., Guangzhou, China) following the manufacturer’s protocol for animal tissues, and stored at -20°C. Most of the voucher specimens and their extracted genomic DNA were deposited in the Insect Collection, Department of Entomology, South China Agricultural University (SCAU).

For molecular markers, we selected two mitochondrial regions (*COI-COII* and *16S*) and one nuclear locus (*EF-1a*), in accordance with Fan *et al*. [1], and added nine new sequences. Moreover, we amplified and sequenced an additional part of the *COI* region using primers LCO1490 and HCO2198. The remainder of the data was obtained from previous studies [1–3] (S1 Table). Methods for the editing and alignment of sequences are detailed in Fan *et al*. [1] and Huang *et al*. [5].

### Phylogenetic analyses

Species of four Baorini genera—*Iton*, *Zenonia*, *Zenonoida*, and *Polytremis*—were selected as outgroup taxa, following Fan *et al*. [1]. The former three genera were closer outgroups, whereas *Polytremis*, sensu Evans, includes all species of *Zinaida* in many works.

Phylogenetic trees were constructed using both maximum likelihood (ML) and Bayesian inference (BI) methods. Prior to these analyses, PartitionFinder V1.1.1 was used to select optimal partitioning scheme [6], and the concatenated dataset was treated with the same partitioning scheme for ML and BI analyses (S2 Table). ML analysis was carried out using IQ-TREE [7] on the online W-IQ-TREE (http://iqtree.cibiv.univie.ac.at/, [8]), and the best-fit models for each partitioned dataset (S2 Table) were selected using the Auto function under the Bayesian Information Criterion (BIC) [9]. We performed 1000 ultrafast bootstrap replicates [10] to evaluate branch support (BP). BI analysis was implemented in MrBayes on XSEDE 3.2.6 [11] using reversible-jump MCMC to allow for sampling across the entire substitution rate models. Four Markov Chains (three heated chains, one cold) were run for 2×10^6^ generations, sampling at every 1000 generations, and discarding the first 25% of sampled trees as burn-in. Bayesian posterior probabilities (PP) were calculated by majority rule consensus from the remaining trees. Tracer 1.6 [12] was used to examine the programs and determine the convergence of the analyses. MrBayes run was carried out on the online CIPRES Science Gateway resource [13]

### Morphology

Examined samples were collected from China, Japan, Indonesia, and Malaysia (see S1 Table and the checklist for details). For the morphological study, we followed the methods in Fan *et al*. [14].

## Results

### Sequence characterization

Twenty-one specimens from five genera were included in this analysis. Twenty-one sequences for *COI-II*, 19 sequences for *EF-1a*, and 17 sequences for *16S* were characterized. The final combined sequences consisted of 1413 *COI-II*, 1066 *EF-1a*, and 527 *16S*. For the dataset, 559 bp sites were variable, and 323 sites were parsimony-informative.

### Phylogenetic analysis

**Fig 1:**
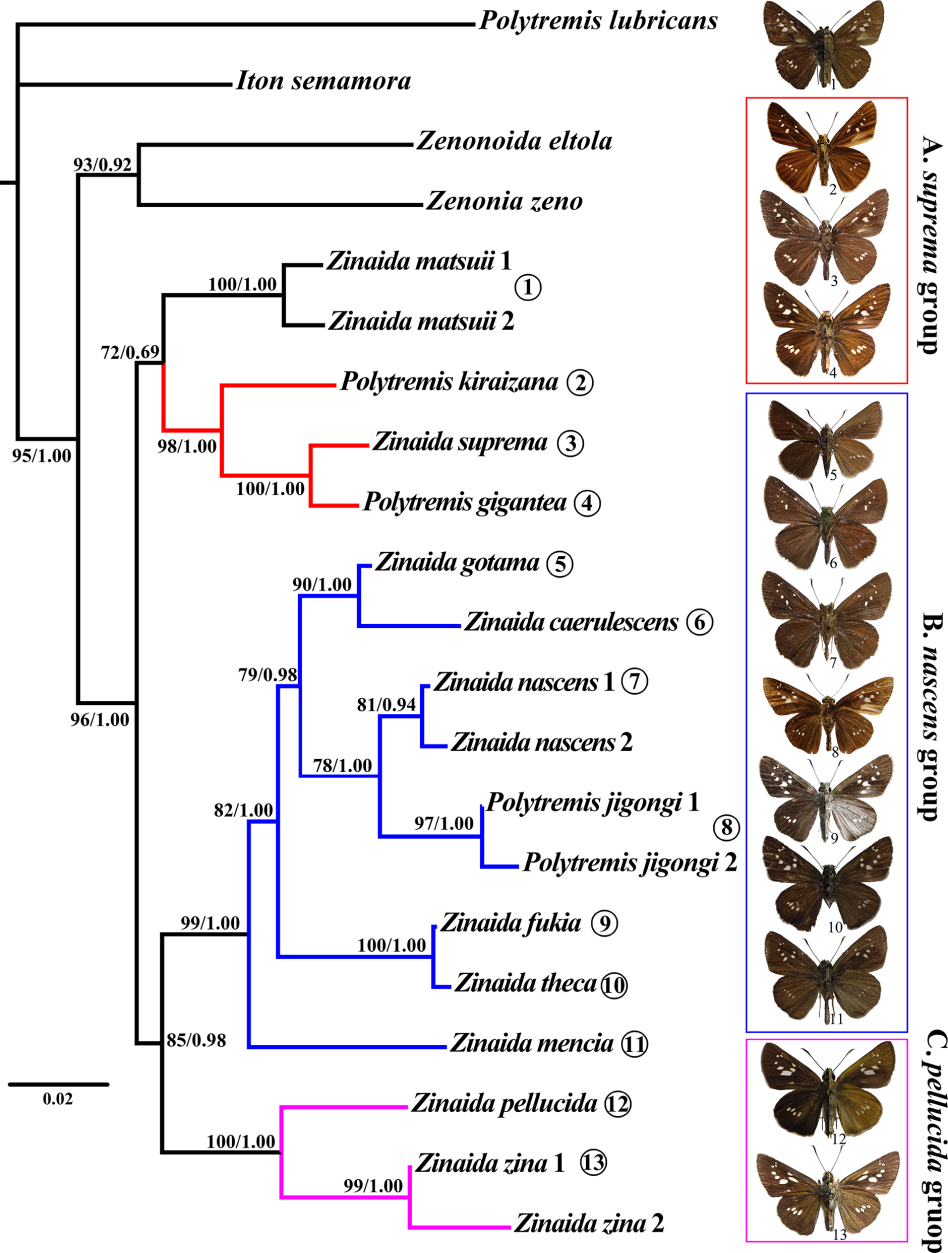
Phylogenetic relationships of the genus *Zinaida* based on the maximum likelihood (ML) analysis of the concatenated *COI-COII*, *16S*, and *EF-1α*. Values at nodes represent the bootstrap support (BP) values of ML analysis and the posterior probabilities (PP) of BI, respectively. The numbers in parentheses refer to the adult photographs.

The same tree topology was obtained using both ML and Bayesian methods (Fig 1). *Polytremis*, as circumscribed by many authors [3, 15–17], is not monophyletic, as shown by Fan *et al*. [1]. The monophyly of the genus *Zinaida* is strongly supported (BP = 96, PP = 1.00). It is divided into four clades, namely the *Z*. *matsuii* clade, Clade A (BP = 98, PP = 1.00), Clade B (BP = 99, PP = 1.00), and Clade C (BP = 100, PP = 1.00). Clade B is sister to Clade C, whereas it is not entirely clear whether the clade comprising *Z*. *matsuii* and Clade A are sisters to each other, or whether one or the other is sister to Clades B + C, due to the lack of strong statistical support (BP = 72, PP = 0.69). Regardless, *P*. *gigantea*, *P*. *jigongi*, *and P*. *kiraizana* were shown to belong to the genus *Zinaida*.

Clade A consists of *Z*. *kiraizana*, *Z*. *suprema*, and *Z*. *gigantea*, and relationships between the three species were well resolved as [*Z*. *kiraizana* + (*Z*. *suprema* + *Z*. *gigantea*)]. Clade C consists of *Z*. *pellucida* and *Z*. *zina*, and is strongly supported in our analyses. The remaining eight species were included in Clade B, and relationships between some of these species were well resolved; for example, *Z*. *gotama* is sister to *Z*. *caerulescens*, and *Z*. *jigongi* is sister to *Z*. *nascens*.

## Discussion

### Phylogeny of the genus *Zinaida*

The phylogenetic framework including nearly all the species of the genus *Zinaida* and its allies revealed four distinct clades that are consistent with their external features, although the relationships between the four groups were not entirely clarified.

**Fig 2:**
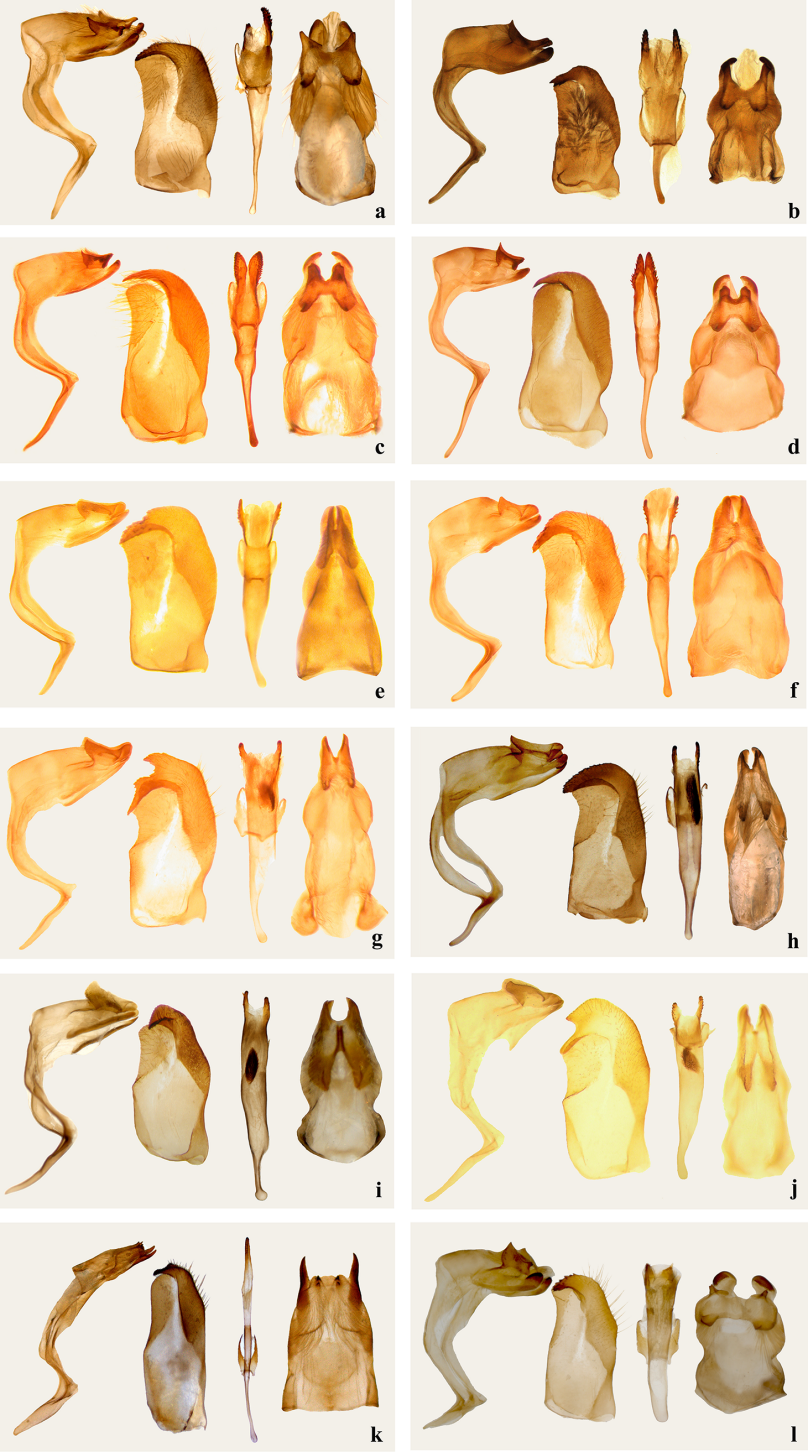
Male genitalia of *Zinaida* (four characteristics, from left to right: Ring, lateral view; valva, inner view; aedeagus; tegumen, dorsal view). a. *Z*. *matsuii*; b. *Z*. *kiraizana*; c. *Z*. *suprema*; d. *Z*. *gigantea*; e. *Z*. *gotama*; f. *Z*. *caerulescens*; g. *Z*. *nascens*; h. *Z*. *jigongi*; i. *Z*. *fukia*; j. *Z*. *mencia*; k. *Z*. *pellucida*; l. *Z*. *zina*.

In this study, the monophyly of the *Z*. *suprema* group (Clade A: *Z*. *suprema*, *Z*. *gigantea*, and *Z*. *kiraizana*) is strongly supported. These species share the following synapomorphies: (1) aedeagus with suprazonal sheath bifurcated into serrated symmetrical processes (processes asymmetric in *Z*. *matsuii*), and (2) cornuti absent (Fig 2A–2D). *Z*. *suprema* is a sister species to *Z*. *gigantea*, and the relationship is supported by overall similarity in male genital characteristics (Fig 2C and 2D). This species group, as well as *Z*. *matsuii*, share the following similar morphological characteristics: (1) forewing with two cell spots, lower one wedge-shaped, pointing toward wing base (Fig 1: 1–4); (2) uncus, left and right projections U-shaped; (3) saccus long and thin; (4) aedeagus, more than basal 2/3 of subzonal sheath thin (Fig 2A–2D). In the analyses of Jiang *et al*. [2], *Z*. *suprema*, *Z*. *gigantea*, *Z*. *kiraizana*, *Z*. *matsuii*, and *Z*. *caerulescens* formed a well-supported clade. However, it is obvious that *Z*. *caerulescens* does not belong to this species group. Fan *et al*. [1] and Zhu *et al*. [3] reclaimed the close association of *Z*. *matsuii* and *Z*. *suprema*, and that of *Z*. *matsuii* and *Z*. *kiraizana*. Interestingly, the three species have a similarly shaped male stigma in space CuA2 on the upper side of the forewing (Fig 1: 1–3). On the basis of our present phylogenetic inference and previous studies, as well as morphological characteristics, we recognize that the *Z*. *suprema* group consists of *Z*. *kiraizana*, *Z*. *suprema*, and *Z*. *gigantea*. For *Z*. *matsuii*, because of the low statistical support and a morphological difference (asymmetric suprazonal sheath of the aedeagus), further study is necessary to confirm its association with this clade.

Fan *et al*. [1] showed a well-supported clade that comprised *Z*. *caerulescens*, *Z*. *nascens*, *Z*. *gotama*, *Z*. *mencia*, *Z*. *theca theca*, and *Z*. *theca fukia*, although interspecies relationships were not clarified. Our phylogenetic inferences confirm that these species form a monophyletic group (Clade B), the *Z*. *nascens* group, within which *P*. *jigongi* is also nested. This analysis also revealed that *P*. *caerulescens* and *Z*. *gotama* are sister species, and placed *P*. *jigongi* as a sister to *Z*. *nascens*. Accepting the conclusion of Jiang *et al*. [4] that *Z*. *theca* and *Z*. *fukia* are distinct species, the *Z*. *nascens* group proposed in this analysis includes the above seven species, which share some notable morphological characteristics: (1) lower cell spot on the forewing not produced toward base, or no spot at all (Fig 1: 5–11); (2) aedeagus with suprazonal sheath bifurcated distally; and (3) uncus V-shaped, left and right projections closer (Fig 2E–2J), and show the following relationships: (*mencia* + ((*theca* + *fukia*) + ((*jigongi* + *nascens*) + (*gotama*+*caerulescens*)))). Morphologically, *Z*. *mencia*, *Z*. *theca*, *Z*. *fukia*, and *Z*. *jigongi* have two cell spots on the forewing, but the remaining three, Z. *nascens*, *Z*. *gotama*, and *Z*. *caerulescens* only have an upper cell spot or no spot. *Z*. *gotama* and *Z*. *caerulescens* have no cornuti, whereas in the remaining five species cornuti are present. Moreover, in *Z*. *theca* and *Z*. *fukia*, the ventrodistal process of the valva is distally short, blunt, and unbifurcated. On the basis of the morphological characteristics detailed in the original description [18], *P*. *micropunctata* belongs to this group and is very probably a sister species of *Z*. *nascens*.

The *Z*. *pellucida* group (Clade C) consists of *Z*. *pellucida* and *Z*. *zina*. A close association between the two species was also identified in previous studies [1–3]. Our morphological study shows that they share the following morphological characteristics: (1) large spots in space M2-CuA1, with the distance between them shorter than in any other species (Fig 1: 12–13); (2) uncus short, left and right projections separated, gnathos far longer than uncus; and (3) aedeagus with suprazonal sheath slightly bifurcated distally or not bifurcated, cornuti absent (Fig 2K, l).

Our analysis lacks these specimens of *Polyremis minuta*, *P*. *annama*, and *P*. *kittii*. On the basis of the morphological characteristics detailed in the original description [15, 19, 20], these species share the following synapomorphies: (1) the third segment of palpi short, stout and barely protruding; (2) forewing cell spots conjoined or only under cell spot present. Therefore, we tentatively assign them to *Zenonoida*. Further study is necessary to confirm this hypothesis, and clarify their phylogenetic placement.

### Distribution

**Fig 3:**
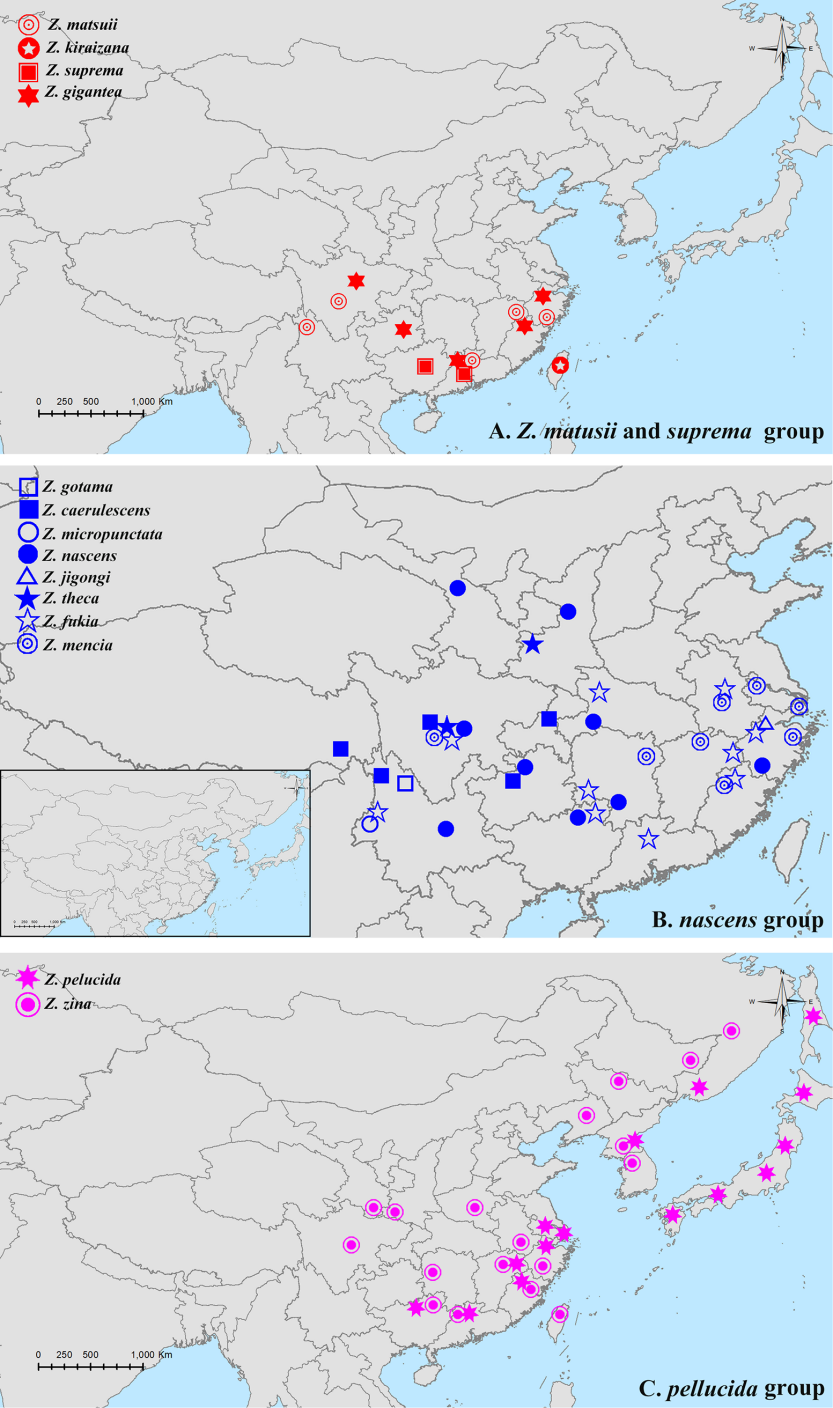
Distribution of *Zinaida*.

Most species in the genus *Zinaida* are endemic to China. Each of the identified clades shows a large degree of similarity in terms of wing pattern and male genitalia characteristics, and exhibits an interesting geographical pattern. The *Z*. *suprema* group and *Z*. *matsuii* have narrower ranges: *Z*. *kiraizana* is only found in Taiwan, whereas the other three species: *Z*. *suprema*, *Z*. *gigantea*, and *Z*. *matsuii* currently occur in Zhejiang, Fujian, Guangdong, Guangxi, Sichuan, Guizhou, and Yunnan [16, 17, 21] (Fig 3A). The sister species *Z*. *suprema* and *Z*. *gigantea* are sympatrically distributed, and have been found simultaneously in Nanling, Guangdong. Conversely, *Z*. *kiraizana* and the former two species (*Z*. *suprema* and *Z*. *gigantea*) have allopatric distributions, suggesting geographical isolation caused by the strait between the Chinese mainland and Taiwan that occurred earlier than the speciation of *Z*. *suprema* and *Z*. *gigantea*.

All species in the *Z*. *nascens* group are endemic to China, and mainly occur south of the Qinling Mountains and east of the Hengduan Mountains (Fig 3B). According to the known distribution data, the center of diversity of the group is central Sichuan (Yaan and Kangding), which is inhabited by five *Zinaida* species: Z. *caerulescens*, *Z*. *nascens*, *Z*. *theca*, *Z*. *fukia*, and *Z*. *mencia*. These species do extend their range to other regions. *Z*. *mencia* and *Z*. *fukia* have the broadest geographical ranges, including southwest China and subtropical (central/east/south) China. In our present analyses, they represent the basal branches of the group. The other species are mainly distributed in southwest China and adjacent regions. Notably, the sister species *Z*. *theca* and *Z*. *fukia*, having been treated as subspecies of *P*. *theca* (Evans, 1949) previously [15], have a narrow geographical overlap in central Sichuan, China [4]. *Z*. *caerulescens* is distributed in Chongqing, Sichuan, Guizhou, Yunnan, and Xizang, and shows an overlap with *Z*. *gotama* in North Yunnan. Interestingly, these two sympatric species are sister species according to the molecular analysis. Clearly, the species are not evenly distributed, and closely follow the distributions of certain topographic features and climatic conditions. The diversity of the group, as well as that of other Chinese skippers, is greatest in Southwest China [22], which is considered one of the world’s biodiversity hotspots because of its meteorological heterogeneity, monsoons, and specific topography, comprising a mosaic of plateaus, mountains, basins, river valleys, and deep gorges [23–25].

Finally, *Z*. *pellucida* and *Z*. *zina* have a broader geographic range than other *Zinaida* species, and have extensive range overlap in Southeastern China, as well as in North Korea (Fig 3C).

### Taxonomic checklist

***Zinaida* Evans**, **1937**

*Zinaida* Evans, 1937: 64 [19]. Type species: *Parana nascens* Leech, 1983.

*Zinaida* Fan *et al*., 2016 [1].

*Diagnosis*.

Medium-sized, brown. Palpi third segment thin, long, prominently protruding; wing semi-hyaline white spots; most males stigma in CuA2 on upper side of forewing. Male genitalia, uncus, left and right projects attached at base, attached to straight gnathos; suprazonal sheath of aedeagus usually bifurcated into serrated processes.

The genus externally very similar to *Polytremis* and *Zenonoida*, can be distinguished from former by wing white spots, undivided lateral process of uncus; and separated from latter by thin, long, prominently protruding third segment, forewing cell spots separated or lower cell spot absent.

**1**. ***Zinaida matsuii* (Sugiyama, 1999)**

*Polytremis matsuii* Sugiyama, 1999: 11–12 [26] (Type locality: Dujiangyan, Sichuan).

*Polytremis matsuii*: Huang, 2002: 114 [27]; Yuan *et al*., 2015: 460 [16]; Wu & Hsu, 2017: 1427 [17].

*Zinaida matsuii*: Fan *et al*., 2016 [1].

Specimens examined: 3♂ (1♂, He484), Hailuogou, Luding County, Sichuan, 2014-VII-4, leg. Zhenfu Huang, Liangliang Li and Wen Fei; 1♂ (1♂, He1050), Longcanggou, Yingjing County, Sichuan, 2016-VII-2, leg. Zhenfu Huang and Jing Tang; 1♂, Wuyishan, Lichuan Country, Jiangxi, 2008-VI.

Distribution: Zhejiang, Jiangxi, Guangdong, Sichuan, Yunnan.

***Z***. ***suprema* group**

**2**. ***Zinaida gigantea* (Tsukiyama, Chiba & Fujioka, 1997) comb. nov.**

*Polytremis gigantea* Tsukiyama, Chiba & Fujioka, 1997: 292 [28] (Type locality: Qingchengshan, Sichan).

*Polytremis gigantea*: Yuan *et al*., 2015: 469[16]; Wu & Hsu, 2017: 1427 [17].

*Polytremis feifei* Huang, 2002: 111 [27]. (Type locality: Qingchengshan, Sichan).

Specimens examined: 1♂, Nanling National Natural Reserve, Ruyuan County, Guangdong, 2003-VII-10, leg. Min Wang; 1♂ (He1052), Longcanggou, Yingjing County, Sichuan, 2016-VII-2, leg. Zhenfu Huang and Jing Tang.

Distribution: Zhejiang, Fujian, Guangdong, Guizhou, Sichuan.

**3**. ***Zinaida kiraizana* (Sonan, 1938) comb. nov.**

*Parnara kiraizana* Sonan, 1938: 255 [29] (Type locality: Taiwan).

*Polytremis mencia kiraizana*: Evans, 1949: 444 [15]; Chou, 1994:730 [30].

*Polytremis kiraizana*: Yamanaka, 1980:126 [31]; Hus, 1989:79 [32]; Chiba *et al*., 2009 [21]: 55; Yuan *et al*. [16], 2015; Wu & Hus, 2017: 1427 [17].

Specimens examined: 2♂, Taizhong City, Taiwan, 1991-VII-29, leg. Yufeng Hsu.

Distribution: Taiwan.

**4**. ***Zinaida suprema* (Sugiyama, 1999)**

*Polytremis suprema* Sugiyama, 1999: 9–10 [26] (Type locality: Dayaoshan, Guangxi).

*Zinaida suprema*: Fan *et al*, 2016 [1].

Specimens examined: 1♂ (He070), Nanling National Natural Reserve, Ruyuan County, Guangdong, 2004-VII-23, leg. Min Wang.

Distribution: Guangdong, Giangxi.

***Z***. ***nascens* group**

**5**. ***Zinaida nascens* (Leech, 1893)**

*Parnara nascens* Leech, 1893: 614 [33] (Type locality: W. China).

*Zinaida nescens*: Evans, 1937: 64 [19]; Fan *et al*, 2016 [1].

*Polytremis nascens*: Evans, 1949 [15]: 444; Bridge, 1994 [34]: 152; Huang *&* Xue, 2004 [35]: 176; Yuan *et al*., 2015 [16]: 456; Wu & Hsu, 2017; 1431 [17].

Specimens examined: 3♂ (1♂, He100), Baoxing county, Sichuan, 2003-VIII-30, leg. Xiaoling Fan and Min Wang; 2♂, Dongan county, Hunan, 2007-VIII, leg. Yiyao Li.

Distribution: Gansu, Shaanxi, Zhejiang, Hubei, Hunan, Guangxi, Guizhou, Sichuan, Yunnan.

**6**. ***Zinaida theca* Evans, 1937**

*Zinaida theca* Evans, 1937: 65 [19] (Type locality: Siao Lou).

*Polytremis theca theca*: Evans, 1949: 445 [15].

*Zinaida theca theca*: Fan *et al*., 2016 [1].

*Polytremis theca*: Jiang *et al*., 2016 [4].

Specimens examined: 3♂, Baoxing county, Sichuan, 2003-VIII-30, leg. Xiaoling Fan and Min Wang; 2♂ (1♂, He503), Jialingjiang, Baoji city, Shaaxi, 2011-VIII-11, leg. Wentang Wang; 1♂, Dabashan, Sichuan, 1996-VI-22.

Distribution: Shaanxi, Sichuan.

**7**. ***Zinaida fukia* Evans, 1940**

**a**. ***Zinaida fukia fukia* Evans, 1940**

*Zinaida theca fukia* Evans, 1940: 230 [34] (Type locality: Fujian).

*Polytremis theca fukia*: Evans, 1949: 445 [15].

[*Polytremis pellucida*: Huang (ed.), 2001: 144.] [36]

*Polytremis fukia*: Jiang (ed), 2016 [4].

*Zinaida theca fukia*: Fan *et al*., 2016 [1].

Specimens examined: 1♂ (He009), Nanling, Guangdong. 2003-VII-13, Min Wang; 5♂1♀, Maoershan, GuangXi, 2003-VII-01, leg. MinWang and Guohua Huang.

Distribution: Zhejiang, Anhui, Hubei, Hunan, Fujian, Jiangxi; Guangdong, Guangxi, Sichuan.

**b**. ***Zinaida fukia macrotheca* (Huang, 2003) comb. nov.**

*Polytremis theca macrotheca* Huang, 2003: 40 [18] (Type locality: Nujiang, Yunnan).

Distribution: Yunnan (Nujiang).

**8**. ***Zinaida gotama* (Sugiyama, 1999)**

*Polytremis gotama* Sugiyama, 1999: 12–14 [26] (Type locality: Zhongdian, Yunnan).

*Zinaida gotama*: Fan *et al*., 2016 [1].

Specimens examined: 1♂ (He010, Fan *et al*., 2016), Luguhu, Yunnan, 2003-VIII-13, leg. Xiaoling Fan and Min Wang; 4♂, 3♀, same data as the former.

Distribution: Yunnan.

**9**. ***Zinaida jigongi* (Zhu, Chen & Li, 2012) comb. nov.**

*Polytremis jigongi* Zhu, 2012 [37] (Type locality: Tianmushan, Zhejiang).

Specimens examined: Paratype: 1♂, Luguhu, Yunnan, 2003-VIII-13, leg. Jianqing Zhu.

Distribution: Zhejiang.

**10**. ***Zinaida micropunctata* (Huang, 2003) comb. nov.**

*Polytremis micropunctata* Huang, 2003: 41–42 [18] (Type locality: Nujiang, Yunnan).

Distribution: Yunnan.

**11**. ***Zinaida mencia* (Moore, 1877)**

*Pamphila mencia* Moore, 1877: 52 [38] (Type locality: Shanghai).

*Zinaida mencia*: Evans, 1937: 65 [19]; Fan, *et al* 2016 [1].

*Polytremis mencia*: Bridges, 1994: 140 [34]; Chou, 1994: 730 [30]; Huang, 2002: 15 [27]; Huang, 2003: 42 [18]; Yuan *et al*., 2015: 459 [16]; Wu & Hsu, 2017: 1427 [17].

Specimens examined: 1♂ (He502), Lushan, Jiangxi, 2007-VII-24, leg. Xiaoling Fan and Min Wang; 1♂, Jiujiang, Jiangxi, 1982-V.

Distribution: Jiangsu, Shanghai, Zhejiang, Anhui, Fujian, Hunan, Jiangxi, Sichuan.

**12**. ***Zinaida caerulescens* (Mabille, 1876)**

*Pamphila caerulescens* Mabille, 1876: 1v [39] (Type locality: Tibet).

*Parnara caerulescens*: Leech, 1893: 615 [33].

*Zinaida caerulescens*: Evans, 1937: 65 [19]; Fan *et al*., 2016 [1].

*Polytremis caerulescens*: Evans, 1949: 444 [15]; Yuan *et al*., 2015: 462 [16]; Wu & Hsu, 2017: 1431 [17].

Specimens examined: 1♂ (He087), Baoxing County, Sichuan, 2003-VII-30, leg. Xiaoling Fan and Min Wang; 1♂, same data as the former.

Distribution: Chongqing, Sichuan, Guizhou, Yunnan, Xizang.

***Z***. ***pellucida* group**

**13**. ***Zinaida pellucida* (Murray, 1874)**

**a**. ***Zinaida pellucida pellucida* (Murray, 1874)**

*Pamphila pellucida* Murray, 1874: 172 [40] (Type locality: Japan).

*Polytremis pellucida pellucida*: Evans, 1949: 445 [15]; Bridges, 1994: 173 [34]; Chou, 1994: 730 [30]; Wang *et al*., 1998: 201[41]; Huang, 2002: 113 [27]; Huang, 2003: 42 [18].

*Zinaida pellucida*: Fan *et al*, 2016 [1].

Specimens examined: 1♂ (He392), Kumamoto, Japan, 2010-IV-31, leg. Hideyuki Chiba; 1♂, Aichi, Seto, Japan, 1999-VIII-13. Leg. Masao Yamanaka.

Distribution: North Korea, Japan, Russia.

**b**. ***Zinaida pellucida quanta* (Evans, 1949)**

*Polytremis pellucida quanta* Evans, 1949: 445 [15] (Type locality: Kwangtsch, Fujian).

Specimens examined: Holotype, ♂, Guadun, Fujian, China, 1937-VII-10, leg. J. Klapperich (BMNH, photos examined, S2 Fig).

Distribution: Fujian.

**c**. ***Zinaida pellucida inexpecta* (Tsukiyama, Chiba & Fujioka, 1997)**

*Polytremis pellucida inexpecta* Tsukiyama, Chiba & Fujioka, 1997 [28] (Type locality: Tianmushan, Zhejiang).

*Polytremis zina*: Tong, 1993: 73, pl. 61, Fig 712–713 [42], Misidentification.

Specimens examined. Holotype, Tianmushan, Zhejiang, China, 1982-V-VII.

Distribution: Jiangsu, Shanghai, Zhejiang, Fujian, Jiangxi, Guangxi, Guangdong.

**14**. ***Zinaida zina* (Evans, 1932)**

**a**. ***Zinaida zina zina* (Evans, 1932)**

*Baoris zina* Evans, 1932: 416 [43] (Type locality: Mount Emei, Sichuan).

*Polytremis zinoides* Evans, 1937: 64 [19] (Type locality: Amur).

*Polytremis zina*: Evans, 1949: 446 [15]; Chou, 1994: 370 [30].

*Polytremis pellucida*: Lee, 1982: 106 [44]; Tong, 1993: 73, pl. 61, Fig 714–715 [42]; Wang & Niu, 1998: 201, pl. 87, Fig 17–18[41]; Wang, 1999: 28 [45]. Misidentification.

*Zinaida zina zina*: Fan *et al*, 2016.

Specimens examined: 1♂ (He037), Nanling National Natural Reserve, Ruyuan County, Guangdong, 2003-V, leg. Xiaoling Fan; 2♂, 2♀, Nanling National Natural Reserve, Ruyuan County, Guangdong, 1997-V, leg. Min Wang; 1♂, Yingde, Guangdong, 2003-VI-13, leg. Guohua Huang; 2♂, Maoershan, Xinan county, Guangxi, 2003-VII-6, leg. Min Wang and Guohua Huang.

Distribution: Heilongjiang, Jilin, Liaoning, Gansu, Shaanxi, Henan, Anhui, Zhejiang, Sichuan, Hunan, Jiangxi, Fujian, Guangxi, Guangdong. North Korea, South Korea, Russia (S. Ussuri).

**b**. ***Zinaida zina asahinai* (Shirôzu, 1952) comb. nov.**

*Polytremis pellucida asahinai* Shirôzu, 1952: 13 [46] (Type locality: Taiwan).

*Polytremis zina taiwana* Murayama, 1981: 10–13 [47] (Type locality: Taiwan).

*Polytremis theca asahinai*: Heppner & Inoue, 1992: 132 [48].

*Zinaida zina taiwana*: Fan *et al*., 2016 [1].

Specimens examined: 1♂ (He545), Taipei, Taiwan, 2014-V; 1♂, Taoyuan, Taiwan, 1988-VI-27, leg. Yufeng Hsu.

Distribution: Taiwan.

## Supporting information

S1 Table List of species used in this study. (DOCX)

S2 Table The best-fit evolutionary partition schemes in PartitionFinder. The best model was selected under a Bayesian Information Criterion (BIC) in W-IQ-TREE.
(DOCX)

S1 Fig *Zinaida nascens* Leech, 1893 (He324, Shannxi). (A) adult; (B) male genitalia.
(TIF)

S2 Fig *Zinaida pellucida quanta* Evans, 1949. (A) upperside; (B) underside.
(TIF)
