# Supplementary material for: Systematics of the genus Zinaida Evans, 1937 (Hesperiidae: Hesperiinae: Baorini)
Source: PLoS One. 2017 Nov 30;12(11):e0188883. doi: 10.1371/journal.pone.0188883 (PMC5708651; doi:10.1371/journal.pone.0188883)
Supplement: S1 Table — (DOCX) [file pone.0188883.s001.docx]

S1 Table. List of species used in this study

| **Species** | **Locality** | **Voucher** | **Accession numbers** | | | **Reference** |
| --- | --- | --- | --- | --- | --- | --- |
|  |  |  | **COI-II** | **EF-1a** | **16s** |  |
| *Zinaida caerulescens* (Mabille, 1876) | China: Sichuan | He087 | JX989090 | KX151616 | JX971172 | Fan et al. 2016 |
| *Zinaida fukia* (Evans, 1940) | China: Guangdong | He009 | JX989092 | KX151629 | JX971174 | Fan et al. 2016 |
| *Zinaida gotama* Sugiyama, 1999 | China: Yunnan | H1-1019 | KT240172 | KT240154 |  | Zhu et al. 2016 |
| *Zinaida matsuii* Sugiyama, 1999 | China: Sichuan | He484 | KX151574 | KX151624 | KX151515 | Fan et al. 2016 |
| *Zinaida matsuii* Sugiyama, 1999 | China: Sichuan | He1050 | **MG012847**  **MG012844** | **MG012841** | **MG012854** | this study |
| *Zinaida mencia* (Moore, 1878) | China: Jiangxi | He502 | **MG012848** |  |  | this study |
|  |  |  | KX151575 | KX151625 | KX151516 | Fan et al. 2016 |
| *Zinaida nascens* (Leech,1893) | China: Sichuan | He100 | JX989094 | KX151626 | JX971176 | Fan et al. 2016 |
| *Polytremis nascens* (Leech, 1893) | China: Shaanxi | He324 | **MG012849**  **MG012845** | **MG012842** | **MG012855** | this study |
| *Zinaida pellucida*  (Murray,1874) | Japan: Kumamota | He392 | KX151576 | KX151627 | KX151517 | Fan et al. 2016 |
| *Zinaida suprema* Sugiyama,1999 | China: Guangdong | He070 | JX989039 | KX151628 | JX971175 | Fan et al. 2016 |
| *Zinaida theca* Evans, 1937 | China: Shaanxi | He503 | **MG012850** |  |  | this study |
|  |  |  | KX151577 | KX151630 | KX151518 | Fan et al. 2016 |
| *Zinaida zina zina* (Evans,1932) | China: Guangdong | He037 | JX989091 | KX151631 | JX971173 | Fan et al. 2016 |
| *Zinaida zina asahinai* Murayama, 1981 | Taiwan | He545 | KX151578 | KX151632 | KX151519 | Fan et al. 2016 |
| *Polytrmis gigantea* Tsukiyama Chiba *&* Fujioka, 1997 | China: Sichuan | He1052 | **MG012851**  **MG012846** | **MG012843** | **MG012856** | this study |
| *Polytremis jigongi* Zhu, 2012 | China: Zhejiang | 121119042 | KC684404 |  |  | Jiang et al. 2013 |
| *Polytremis jigongi* Zhu, 2012 | China: Zhejiang | 121119043 | KC684405 |  |  | Jiang et al. 2013 |
| *Polytremis kiraizana* (Sonan,1938) | Taiwan | H1-1437 | KT240173 | KT240155 |  | Zhu et al. 2016 |
| *Polytremis* *lubricans* (Herrich-Schäffer, 1869) | China: Hainan | He160 | **MG012852** |  |  | this study |
|  |  |  | JX989089 | KX151620 | JX971171 | Fan et al. 2016 |
| *Zenonoida eltola* (Hewitson, 1869) | China: Hunan | He509 | **MG012853** |  |  | this study |
|  |  |  | KX151571 | KX151618 | KX151511 | Fan et al. 2016 |
| Zenonia zeno (Trimen, 1864) | Cameroon: N. Cameroon | SZA-ZEN-001 | KX151580 | KX151635 | KX151521 | Fan et al. 2016 |
| *Iton* semamora (Moore, 1988) | Idonesia: Sumatra | He239 | JX989109 | KX151607 | JX971192 | Fan et al. 2016 |
