# Supplementary material for: Systematics of the genus Zinaida Evans, 1937 (Hesperiidae: Hesperiinae: Baorini)
Source: PLoS One. 2017 Nov 30;12(11):e0188883. doi: 10.1371/journal.pone.0188883 (PMC5708651; doi:10.1371/journal.pone.0188883)
Supplement: S2 Table — The best model was selected under a Bayesian Information Criterion (BIC) in W-IQ-TREE. (DOCX) [file pone.0188883.s002.docx]

**S2 Table. The Best-fit evolutionary partition schemes in PartitionFinder. The best model was selected under a Bayesian Information Criterion (BIC) in W-IQ-TREE.**

| **subset** | **Scheme of partition** | **Partitions** | **Best-fitting model** |
| --- | --- | --- | --- |
| 1 | GeneCOII_pos3, GeneCOI_pos1 | 1-1190\3, 1260-1413\3 | TIM+I+G |
| 2 | GeneCOII_pos2, GeneCOI_pos2, GeneEF_pos3, GeneT-RNA | 2-1190\3, 1191-1257, 1259-1413\3, 1942-3005\3 | TrN+I |
| 3 | GeneCOI_pos3 | 3-1190\3 | HKY+G |
| 4 | Gene16S, GeneCOII_pos1 | 1258-1413\3, 1414-1939 | TVM+I+G |
| 5 | GeneEF_pos1 | 1940-3005\3 | TVM+G |
| 6 | GeneEF_pos2 | 1941-3005\3 | TrN |
